# PD-1 inhibitor combined with radiotherapy and GM-CSF in MSS/pMMR metastatic colon cancer: a case report

**DOI:** 10.3389/fonc.2023.1078915

**Published:** 2023-04-28

**Authors:** Jiabao Yang, Pengfei Xing, Yuehong Kong, Meiling Xu, Liyuan Zhang

**Affiliations:** ^1^ Department of Radiotherapy & Oncology, The Second Affiliated Hospital of Soochow University, Suzhou, China; ^2^ Institution of Radiotherapy & Oncology, Soochow University, Suzhou, China; ^3^ Laboratory for Combined Radiotherapy and Immunotherapy of Cancer, The Second Affiliated Hospital of Soochow University, Suzhou, China

**Keywords:** colorectal cancer, immunotherapy, radiotherapy, GM-CSF, MSS/pMMR, case report

## Abstract

Patients with chemo-refractory metastatic colorectal cancer (mCRC) have poor prognoses. The application of programmed cell death protein 1 (PD-1)/programmed cell death ligand 1 (PD-L1) inhibitors encouragingly improved the survival of mCRC patients with microsatellite instability-high (MSI-H)/mismatch repair-deficient (dMMR). Unfortunately, it was ineffective for mCRC with microsatellite-stable (MSS)/proficient mismatch repair (pMMR), which accounted for 95% of mCRC. Radiotherapy can promote local control by directly killing tumor cells and inducing positive immune activities, which might help synergistically with immunotherapy. We present the report of an advanced MSS/pMMR mCRC patient who had progressive disease (PD) after first-line chemotherapy, palliative surgery and second-line chemotherapy combined with targeted therapy. Then the patient received the therapy of PD-1 inhibitor combined with radiotherapy and granulocyte-macrophage colony-stimulating factor (GM-CSF). According to Response Evaluation Criteria in Solid Tumors version 1.1 (RECIST1.1), the patient showed a complete response (CR) after triple-combined therapy with progression-free survival (PFS) for more than 2 years so far. The patient had no other significant adverse reactions except for fatigue (Grade 1). The triple-combination therapy provided a promising strategy for metastatic chemo-refractory MSS/pMMR mCRC patients.

## Introduction

By 2021, colorectal cancer (CRC) was the third most common cause of cancer mortality worldwide. Meanwhile, metastasis was found at the first diagnosis in 20% of CRC patients (1). Although the United States Food and Drug Administration (FDA) has approved PD-1 inhibitor pembrolizumab for the treatment of microsatellite instability-high (MSI-H) metastatic CRC (mCRC), about 95% of mCRC patients are MSS/pMMR and cannot benefit from PD-1 inhibitor monotherapy (2). The clinical trials of KEYNOTE-016 and KEYNOTE-028 showed no response in MSS mCRC patients treated with pembrolizumab (3, 4). The preclinical studies of MSS colorectal cancer mice models have shown synergy between radiotherapy and anti-PD-1 in modulating anti-tumor immune responses (5, 6). It provides a theoretical basis for the combination of radiotherapy and immunotherapy. Recently, several I/II clinical studies have shown that the combination of radiotherapy and immunotherapy could improve clinical outcomes in mCRC patients with MSS/pMMR with acceptable toxicity (7–9). Granulocyte-macrophage colony-stimulating factor (GM-CSF), known as an immunomodulatory cytokine, might improve the efficacy of immunotherapy in advanced biliary cancers (10). It is necessary to explore novel strategies for treating MSS/pMMR mCRC, and combining anti-PD-1 immunotherapy with radiotherapy and GM-CSF therapy might be a potential one.

We present the report of a refractory mCRC patient with MSS/pMMR who received PD-1 inhibitor combined with Radiotherapy and GM-CSF. The patient demonstrated a sustained tumor response and prolonged progression-free survival (PFS) for over 2 years so far.

## Case presentations

**Figure 1:**
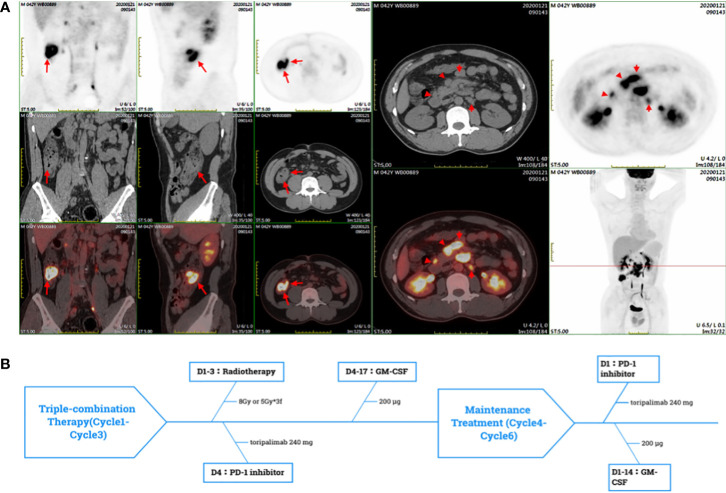
**(A)** The PET-CT showed the high SUV_mean_ of ascending colon and enlarged lymph nodes of retroperitoneal and peritoneal. **(B)** In a PRaG cycle, radiotherapy was delivered for metastases, followed by GM-CSF subcutaneous (sc) injection once daily for two weeks, and toripalimab was intravenous(iv) once within one week after radiotherapy. PRaG Therapy was repeated every three weeks, and three cycles of triple-therapy were administered. Subsequently, the patient underwent three cycles of PD-1 inhibotor and GM-CSF maintenance treatment.

A patient in his mid 40s was diagnosed with ascending colon adenocarcinoma through colonoscopy biopsy and pathological examination on 21 January 2020. Further Positron Emission Tomography/Computed Tomography (PET/CT) imaging showed metastasis of retroperitoneal and celiac lymph nodes (**Figure 1A**). According to the AJCC 8th TNM staging system, the patient was staged T3N2bM1a (c-Stage IVA). Immunohistochemical (IHC) staining was AE1/AE3(+), MSH2 (+), MSH6 (+), MLH1 (+), PMS2 (+), HER-2 (0), PD-L1 (+,CPS=10), CD8 (+,15%), CD68 (+,80%) (**Supplementary Figure 1**). Genetic testing of tumor tissue revealed that missense mutation A146T was found in exon 4 of the KRAS gene. No mutation was found in the BRAF/NRAS. The microsatellite state detection showed a microsatellite-stable (MSS) phenotype by Next Generation Sequencing (NGS).

Considering that the patient was young and was willing to receive surgery, two cycles of conversion therapy of mFOLFOX4 were administered from January to February 2020. Bevacizumab was not added to the conversion therapy, which may cause surgical complications such as bleeding and gastrointestinal perforation. After the conversion chemotherapy, the patient underwent palliative surgery (R2 resection) in February 2020. All of the primary lesion and the part of the mesenteric lymph nodes were resected. The mesenteric and retroperitoneal lymph nodes were not resected. Then the patient received six cycles of chemotherapy of capecitabine plus irinotecan (mXELIRI) combined with Bevacizumab from March to July 2020. CT scan observed new lymph node metastases at mesenteric and retroperitoneal in August 2020, which indicated progressive disease (PD). It suggested the patient was insensitive to chemotherapy combined with vascular-targeted Therapy. In addition, neutropenia and gastrointestinal reaction (Grade 2) were observed during chemotherapy.

**Figure 2:**
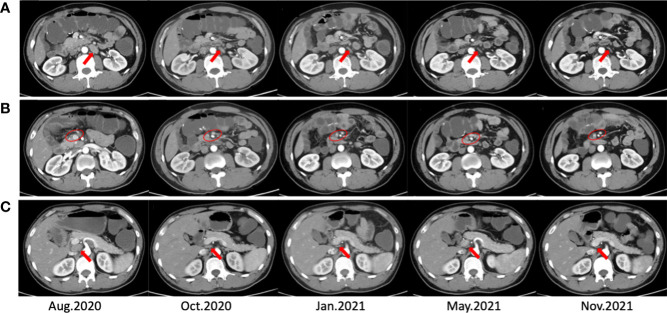
CT scans before, during, and after the PRaG therapy. The CT scans **(A, B)** showed shrunk and disappeared of irradiated lymph node metastases. The CT scans **(C)** showed shrunk and disappeared of nonirradiated lymph node metastases. The arrows point to individual lymph nodes, and the circles include fusion lymph nodes.

**Figure 3:**
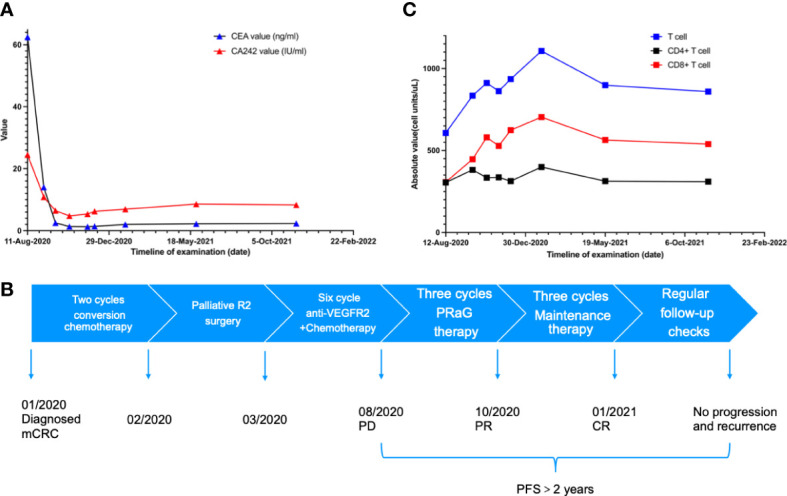
**(A)** The carcinoembryonic antigen (CEA) dropped to the normal range after two cycles of PRaG Therapy. In the whole course of treatment, the patients had no obvious adverse reactions. Due to the influence of COVID-19, the re-examination interval was longer than expected. **(B)** The scheme shows the complete treatment process of the patient. **(C)** The number and activation of lymphocytes are related to the efficacy of immunotherapy.

Considering the side effects of chemotherapy, the patient refused to continue the chemotherapy. Then the patient was enrolled in a prospective phase II clinical trial which was conducted to assess the clinical efficacy and safety of PD-1 (toripalimab) inhibitor combined with Radiotherapy and GM-CSF (Recombinant Human Interleukin-2(I) for Injection) in patients with advanced metastatic solid tumors on 10 August 2020 (ChiCTR1900026175, http://www.chictr.org.cn/index.aspx). We defined the triple-combined therapy of PD-1 inhibitor combined with Radiotherapy and GM-CSF as PRaG therapy. The patient underwent three cycles of PRaG therapy in August 2020 and September 2020. In the PRaG cycle, radiotherapy (8Gy or 5Gy/d, d1-3) was delivered for lymph node metastases, followed by subcutaneous injection of GM-CSF (200μg once daily, d4-17) and intravenous injection of toripalimab (240mg, d4). PRaG regimen was repeated every three weeks (**Figure 1B**). After the 3 cycles of PRaG therapy, the patient achieved partial response (PR) according to RECIST1.1 by CT scan. CT showed a significant decrease of irradiated lymph node metastases in retroperitoneal and celiac (**Figures 2A, B**), and a reduction of nonirradiated lymph node metastases in celiac was also observed (**Figure 2C**). Moreover, tumor markers of carcinoembryonic antigen (CEA) and carbohydrate antigen 242 (CA242) decreased to normal range after two cycles of PRaG therapy (**Figure 3A**).

Three different metastatic sites were chosen for irradiation in three cycles. In the first two cycles, radiotherapy (8Gy/d, d1-3) was delivered for celiac metastatic lymph node and retroperitoneal metastatic lymph node. Considering the tolerance dose constraints of normal tissues, the radiation dose was reduced to 5Gy/d (d1-3) for celiac lymph node in the third cycle.

After the PRaG therapy, the CT scan showed that lymph node metastases in retroperitoneal and celiac almost disappeared compared with before (**Figure 2**). The clinical response of PRaG therapy was complete remission (CR) based on RECIST1.1. Due to the significant decrease of the lesions, no lesions can be irradiated in the follow-up treatment. Subsequent maintenance therapy was implemented with toripalimab and GM-CSF for three cycles from October 2020 to January 2021. The patient exhibited no tumor progression or recurrence in the next two re-examinations by CT scan. The patient had no other significant adverse reactions except for fatigue (Grade 1). The PFS has more than 2 years so far (**Figure 3B**). Due to the influence of COVID-19, the re-examination interval was longer than expected. The recent follow-up in August 2022 showed that the patient maintained a good physical condition and exhibited sustained CR.

## Discussion

Backline treatment of MSS mCRC is a hard nut to crack. Regorafenib or trifluridine and tipiracil (TAS-102) were recommended, but the survival benefit was still limited. A phase II trial of TAS-102 combined with nivolumab showed no tumor response in MSS/pMMR mCRC (11). The mPFS was 2.8 months (95% CI,1.8 to 5.1). 72% of the patients experienced grade grade ≥3 adverse events (AEs). The REGONIVO study from Japan showed that the combination of regorafenib and nivolumab achieved encouraging results in treating MSS mCRC (12). The mPFS was 7.9months (95%CI,2.9 to not reached [NR]), but outcomes of this trial were not reproduced in subsequent clinical studies. These clinical trials showed that the existing combined therapies were limited in overcoming the immune resistance of MSS/pMMR mCRC. A recent study revealed that MSS mCRC patients might benefit from the combination of radiotherapy, anti-PD-1 and anti-CTLA-4 immunotherapy (8). It is necessary to develop suitable combination strategies to improve the efficacy of immunotherapy for the MSS/pMMR mCRC.

Radiotherapy is an essential local tumor control treatment method. In recent years, studies have found that adding radiotherapy can enhance the anti-tumor effect of immunotherapy. Radiation can transform tumor cells into *in-situ* vaccines that can promote tumor cells to release tumor-associated antigens (TAAs) and induce immunogenic cell death (ICD) (13). The PEMBRO-RT study has reported the synergistic effect between immunotherapy and radiotherapy in advanced metastatic NSCLC. The ORR of combined therapy was 36% vs. 18% of the control group (pembrolizumab alone). The combined therapy’s mPFS and mOS were better than the control group (mPFS: 6.6 vs. 1.9 months, mOS: 15.9 vs. 7.6 months). The adverse reactions between the combined and control groups have no significant difference (14). The same results were obtained in PEMBRO-RT and MDACC clinical trials pooled analysis. The combined therapy prolonged the mPFS and mOS than the pembrolizumab alone in patients with metastatic NSCLC (PFS: 9.0 vs. 4.4 months, median OS: 19.2 vs. 8.7 months) (15).

GM-CSF is a cytokine used to promote the growth of leukopenia or neutropenia and is widely used to promote the production of granulocytes or APCs. Preclinical studies supported that GM-CSF combined with immune checkpoint inhibitors (ICI) can improve the activity of innate immune cells, and indirectly recruit T cells by promoting the antigen cross-presentation (16, 17). Ipilimumab combined with GM-CSF can prolong the OS of advanced melanoma more than ipilimumab alone (mOS: 17.5 vs. 12.7 months) (18). In addition, a prospective clinical study has shown that local radiotherapy combined with GM-CSF can improve the prognosis of patients with advanced metastatic solid tumors (19).

The doses and frequency of radiation have not been standardized when radiotherapy is combined with PD-1/PD-L1 inhibitors. Preclinical studies have shown that hypo-fractionated radiotherapy (5Gy × 3f) boosted more proliferation and activation of antigen-presenting cells compared with conventionally fractionated radiotherapy (2Gy × 5f) (20). The conventional fraction also caused more lymphocyte death than hypo-fraction regimens, which affects the response to immunotherapy (21). When the fraction dose of radiation exceeds 5Gy, radiotherapy can indirectly promote the ICD of the tumor (22). However, a higher fraction dose did not represent a better response for treatment. Studies have shown that the increase of Tregs will offset the local control effect when therapy with a single high dose (15Gy × 1f), while the medium fraction regimen (7.5-10Gy × 2-3f) can maintain the low level of Treg and activate the immune response effectively (23). In the PEMBRO-RT study, the fraction regimen (8Gy × 3f) combined with PD-1 inhibitors had excellent clinical efficacy in advanced metastatic NSCLC (14). No additional adverse reactions of immunotherapy were added at this fraction dose. The fraction regimen (3×8Gy or 3×5Gy) we used in PRaG Therapy could be a reasonable choice.

Considering the heterogeneity of the tumor, irradiation of a single site may not induce sufficient exposure to TAAs. Chang et al. suggested multisite radiotherapy of metastases to enhance the synergistic effect (24). However, multisite irradiation may increase the volume of irradiation and lead to a higher incidence of adverse reactions. The number and activation of lymphocytes are related to the efficacy of immunotherapy (25). The decrease in lymphocyte number caused by lymph node irradiation directly reduces the efficacy of immunotherapy (26). Considering side effects and lymphocyte depletion caused by irradiation, it is difficult to irradiate all sites in one cycle for patients with large masses and multiple metastases. Therefore, we chose one lesion to irradiate each cycle. The treatment consists of multiple cycles. Compared to conventional radiotherapy, the range of irradiated lesions was smaller and the total radiation dose was lower (**Supplementary Figures 2–4**). We suggested multiple cycles of radiotherapy, with each cycle targeting a small volume that might protect the lymphocytes and produce sustained immune activation (27). Moreover, irradiation of the Tumor-draining lymph nodes (TDLNs) can benefit patients with lymph node metastases. In this case, there was no significant decrease in lymphocytes, which may be one reason for the excellent efficacy (**Figure 3C**).

After three irradiation cycles achieved an excellent local control effect with a significant decrease in the irradiated lymph node metastases. At the same time, the regression of the nonirradiated lesion was also observed. Regression of the nonirradiated tumor was called the abscopal effect, which was not frequently in patients with radiotherapy alone (28, 29). However, we cannot be sure that the regression of the nonirradiated lesion was caused by abscopal effect in this case. The regression of nonirradiated lesions might be due to the sensitization of radiotherapy or GM-CSF to immunotherapy. Moreover, MSS CRCs were divided into multiple subtypes by consensus molecular subtypes (CMS) consortium. Most of MSS CRCs are immune desert and has no immune cell infiltration, a small portion of them do have CD8+ cell infiltration but suppressed by TME (30). According to the results of IHC and NGS, the immune phenotype of this patient was low CD8+ cell infiltrated. We think that only a subgroup of MSS mCRCs should be considered for this combinational therapy of anti-PD1, radiotherapy and GM-CSF. The specific mechanisms remain to be further studied.

## Conclusions

The MSS mCRC patient achieved terrific results through PRaG triple-combination therapy with well-tolerated. The efficacy and safety of PRaG therapy for MSS mCRC patients need to be confirmed in future prospective studies.

## Data availability statement

The original contributions presented in the study are included in the article/**Supplementary Material**. Further inquiries can be directed to the corresponding author.

## Ethics statement

The studies involving human participants were reviewed and approved by the Ethics Committee of the Second Affiliated Hospital of Soochow University. The patients/participants provided their written informed consent to participate in this study. Written informed consent was obtained from the individual(s) for the publication of any potentially identifiable images or data included in this article.

## Author contributions

JY composed the manuscript as the first authorship. LZ designed and conducted the study as corresponding authors. PX and YK helped with data collection and interpretation. MX collected and sorted out part of the image data. All authors read and approved the final manuscript.
