# Supplementary figures and images for: PD-1 inhibitor combined with radiotherapy and GM-CSF in MSS/pMMR metastatic colon cancer: a case report
Source: Front Oncol. 2023 Apr 28;13:1078915. doi: 10.3389/fonc.2023.1078915 (PMC10176449; doi:10.3389/fonc.2023.1078915)

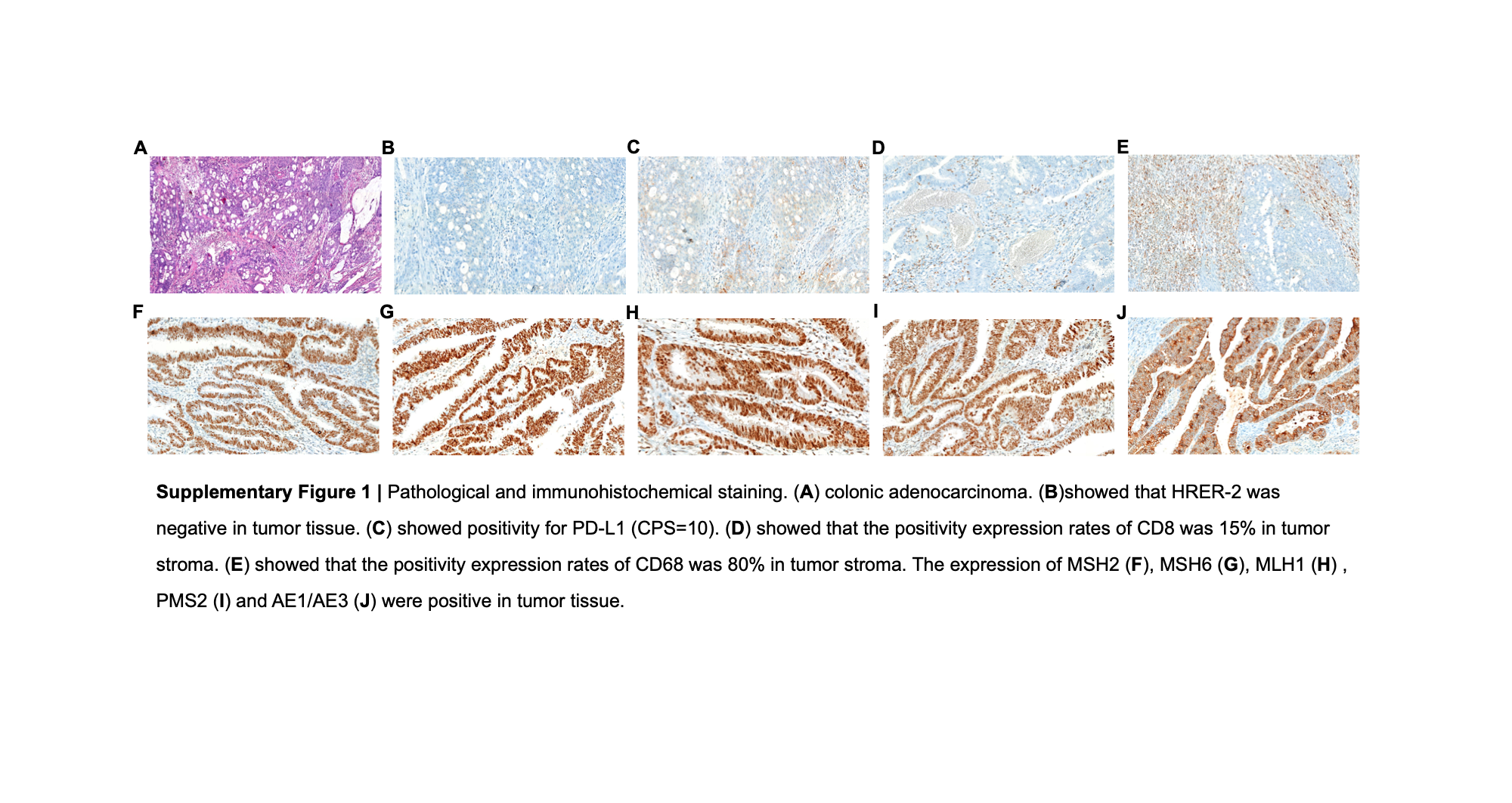

Supplement: Supplementary file 1 [file Image_1.tiff]

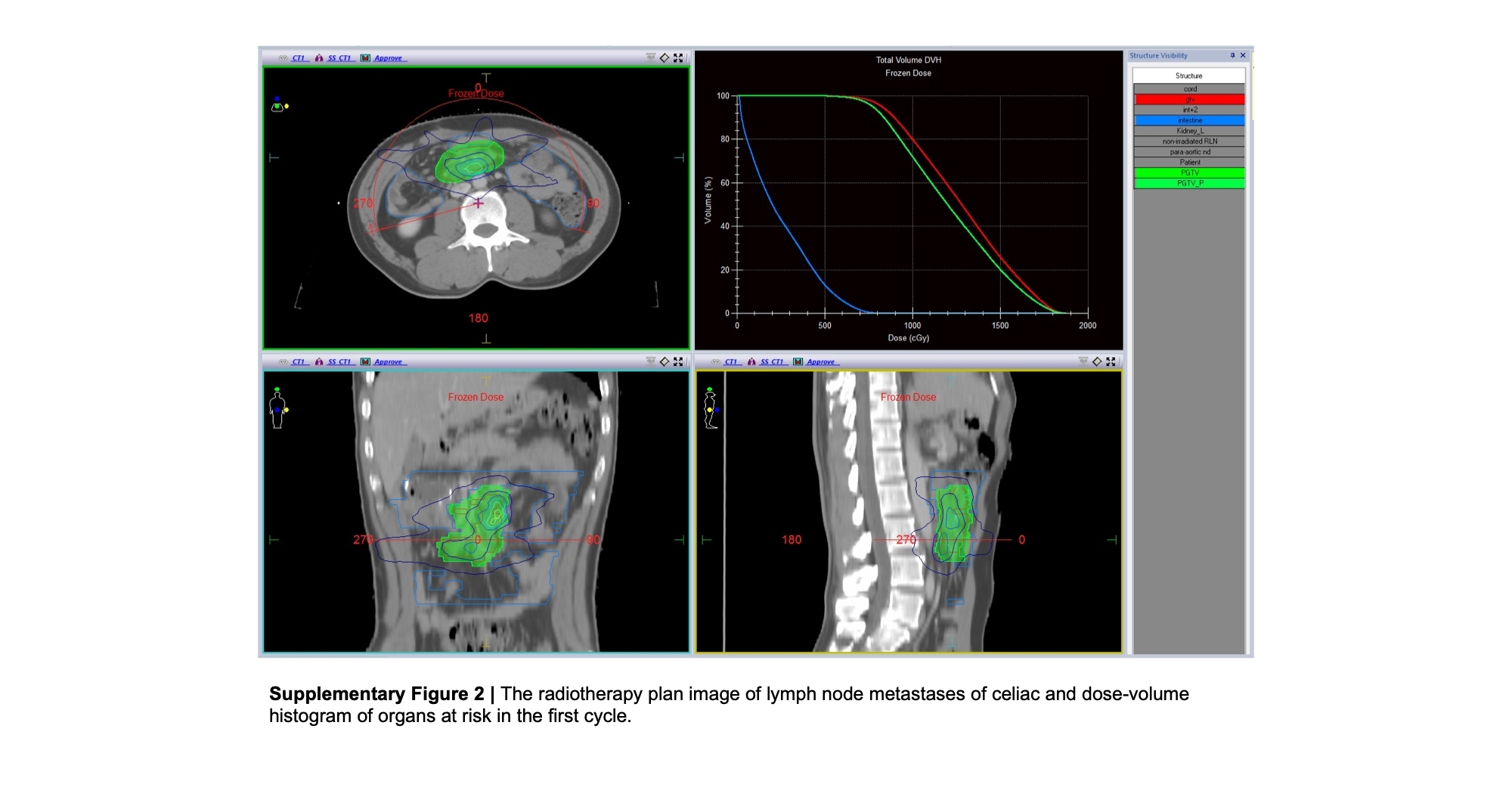

Supplement: Supplementary file 2 [file Image_2.tiff]

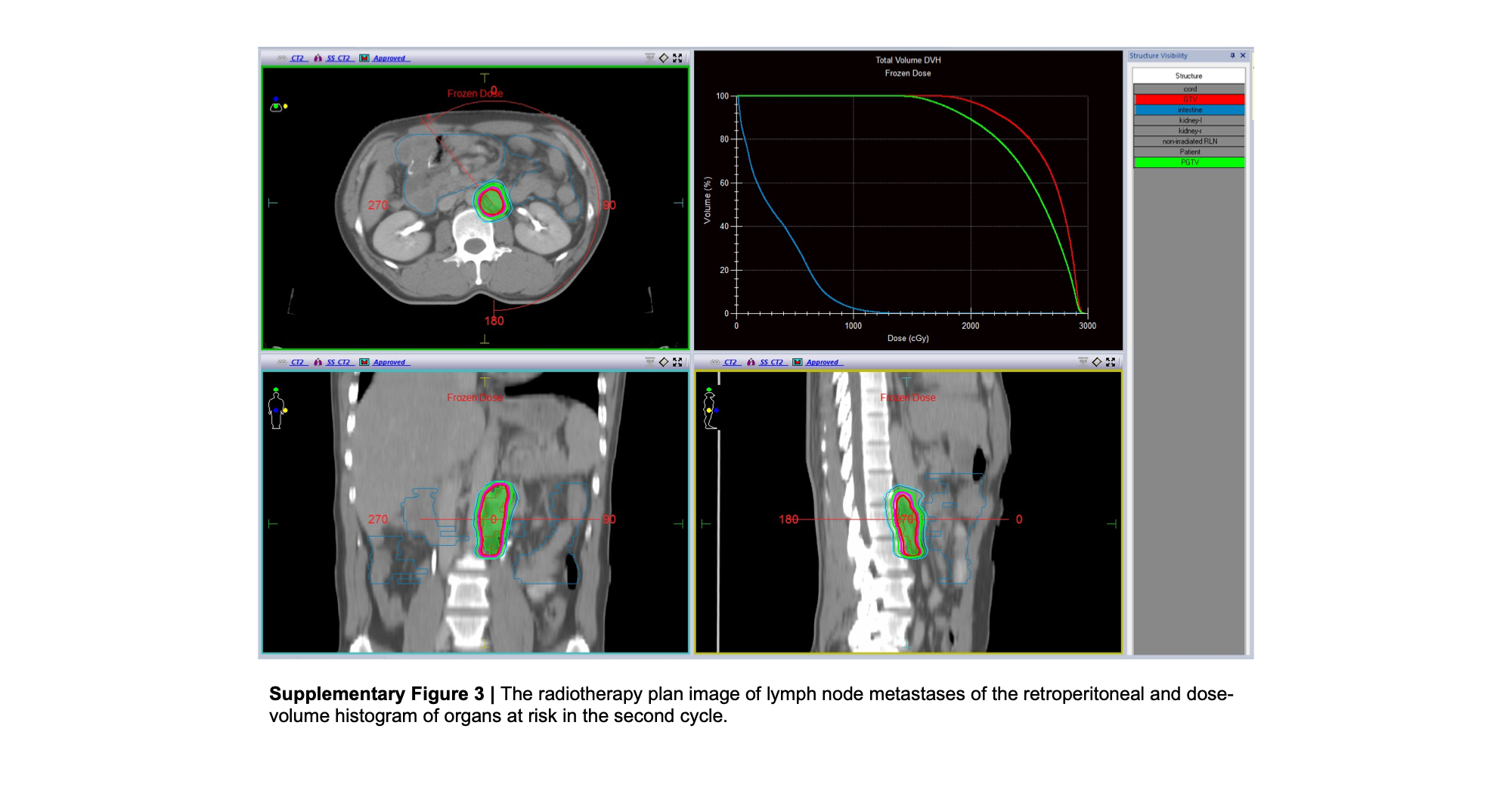

Supplement: Supplementary file 3 [file Image_3.tiff]

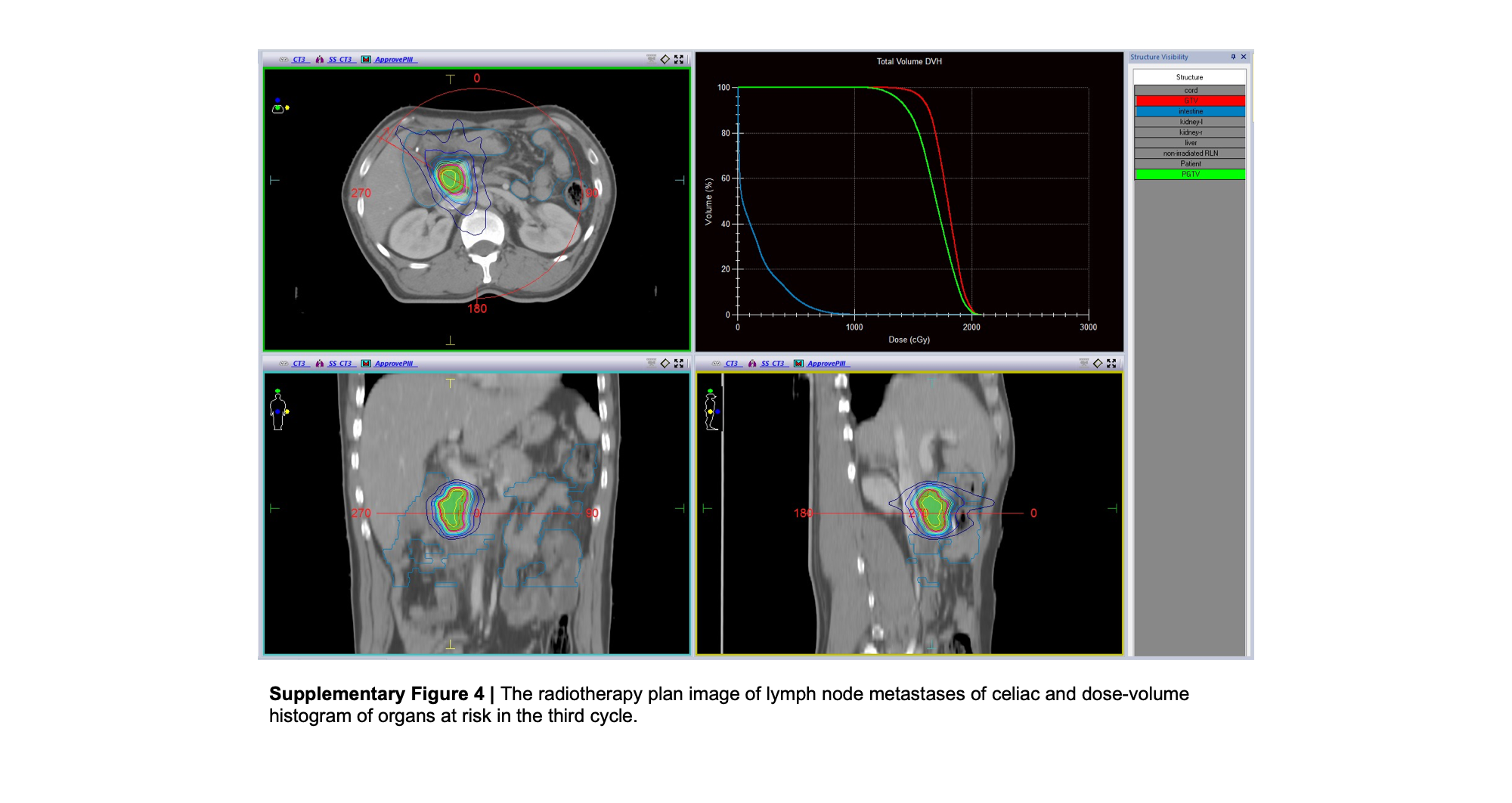

Supplement: Supplementary file 4 [file Image_4.tiff]
